# Hämoadsorption zur Blutreinigung – Unvergleichbarkeit der klinisch angebotenen Verfahren

**DOI:** 10.1007/s00063-020-00702-2

**Published:** 2020-06-24

**Authors:** C. G. Krenn, H. Steltzer

**Affiliations:** 1 Klinik für Anästhesie, Allgemeine Intensivmedizin und Schmerztherapie, Medizinische Universität Wien, Währinger Gürtel 18–20, 1090 Wien, Österreich; 2 Abteilung für Anästhesiologie und Intensivmedizin, Traumazentrum Wien, Standort Meidling, Meidling, Österreich; 3 Lehrstuhl für Intensivmedizin, Sigmund Freud Privatuniversität Wien, Wien, Österreich

**Keywords:** Hämoadsorption, Blutreinigung, Adsorbertechnologien, Hyperinflammation, Sepsis, Hemoadsorption, Blood purification, Adsorber technologies, Hyperinflammation, Sepsis

## Abstract

**Hintergrund:**

Im Bereich der Intensivmedizin, aber auch verstärkt in der Herzchirurgie, gewinnt der Einsatz von adsorptiven Blutreinigungstechnologien zur Behandlung von hyperinflammatorischen Zuständen an Bedeutung. Neben dem CytoSorb-Verfahren, das zunehmend klinisch akzeptiert und auch aktuell am häufigsten eingesetzt wird, drängen seit Kurzem weitere Firmen – vor allem aus China – mit ähnlichen Konzepten auf den Markt.

**Ziel der Arbeit:**

Vor diesem Hintergrund soll es Ziel dieses Artikels sein, exemplarisch auf unterschiedliche Aspekte zwischen den am Markt angebotenen Hämoadsorptionsprodukten einzugehen und einen kritischen Blick auf die vorhandene Evidenz zu werfen.

**Methoden:**

Analysiert wurden technische Merkmale, applikationsspezifische Besonderheiten und vorhandene Evidenz der Adsorptionstechnologien CytoSorb® (CytoSorbents^TM^ Inc., Monmouth Junction, NJ, USA), Jafron® HA-Serie (Jafron Biomedical Co., Guangdong, China) sowie der Biosky® MG-Serie (Biosun® Medical Technology Co., Foshan City, Guangdong Province, China). Nicht einbezogen wurden rein substanzspezifische Verfahren zur Endotoxinelimination (Toraymyxin®, Alteco®).

**Ergebnisse:**

Bei umfassender Analyse dieser Kriterien zeigt sich, dass zwischen den verfügbaren Technologien erhebliche Unterschiede hinsichtlich verwendeter Materialien, Adsorptionscharakteristika, Anwendung und verfügbarer Daten zu Sicherheit und klinischer Erfahrungen bestehen. Darüber hinaus wird deutlich, dass bei Blutreinigungstechnologien nicht nur deren Wirksamkeit unter Berücksichtigung eines Effekt-Preis-Leistungs-Verhältnisses betrachtet werden sollte, sondern insbesondere auch die klinische Sicherheit der einzelnen Verfahren von entscheidender Bedeutung ist.

**Diskussion:**

Unter den analysierten Technologien stellt unserer Ansicht nach derzeit CytoSorb das am besten untersuchte und klinisch am weitesten etablierte Verfahren dar. Darüber hinaus ist darauf hinzuweisen, dass klinische Ergebnisse, insbesondere jedoch auch sicherheitsrelevante Aspekte, aufgrund der technisch unterschiedlichen Verfahren nicht zwischen den Produkten übertragbar sind.

## Abstract (en)

**Background:**

In the field of intensive care medicine, but also increasingly in cardiac surgery, the use of adsorptive blood purification technologies for the treatment of hyperinflammatory conditions is becoming progressively more important. In addition to the CytoSorb concept, which is more and more clinically accepted and currently the most frequently used method, other companies—particularly from China—have recently entered the market with similar concepts.

**Objectives:**

Given this, the aim of this article is to analyze the different aspects of the various hemoadsorption products offered on the market today and to take a critical look at the available evidence.

**Methods:**

Technical features, application-specific characteristics, and the existing evidence of the adsorption technologies CytoSorb® (CytoSorbents^TM^ Inc., Monmouth Junction, NJ, USA), Jafron® HA series (Jafron Biomedical Co., Guangdong, China), and Biosky® MG series (Biosun® Medical Technology Co., Foshan City, Guangdong Province, China) were analyzed. The purely substance-specific methods for endotoxin elimination only (Toraymyxin®, Alteco®) were not considered.

**Results:**

A comprehensive analysis of these criteria reveals that there are considerable differences between the various available technologies in terms of materials used, adsorption characteristics, application, and available data on safety and clinical experience. Furthermore, it becomes clear that not only the efficacy of blood purification technologies should be considered in terms of an effect–price–performance ratio, but that in particular the safety of the individual technologies is of crucial importance.

**Discussion:**

Among the technologies analyzed, CytoSorb currently represents the most investigated and clinically established procedure. Furthermore, it should be noted that clinical results, but particularly safety-relevant aspects, are not transferable between the products due to technically different procedures.

Im Bereich der Intensivmedizin, aber auch verstärkt in der Herzchirurgie, gewinnt der Einsatz von Blutreinigungstechnologien zur Behandlung von hyperinflammatorischen Zuständen wieder an Bedeutung. Hämoadsorptionstechnologien für den Einsatz im septischen Schock, aber auch bei anderen Krankheitsbildern, bei denen eine Reduktion inflammatorischer Mediatoren sinnvoll erscheint, stehen hier im Vordergrund. In Europa wurde dafür in den letzten Jahren das CytoSorb-Verfahren zugelassen, das klinisch zunehmend akzeptiert ist und mit wachsender Häufigkeit zum Einsatz kommt. Daneben ist jedoch zu beobachten, dass nun seit Kurzem weitere Firmen – vor allem aus China – mit ähnlichen, stark angelehnten Konzepten auf den Markt drängen.

Obwohl das physikalische Prinzip der verfügbaren Verfahren zunächst ähnlich erscheint und der Eindruck entstehen mag, dass die in der Literatur verfügbaren labordiagnostischen sowie klinischen Daten unmittelbar auf jede dieser Technologien übertragbar wären, lohnt ein Blick auf die unterschiedlichen technischen Merkmale und als auch applikationsspezifische Besonderheiten der verschiedenen Adsorptionstechnologien und ein kritischer Blick auf vorhandene wissenschaftliche Evidenz. Dabei soll es in diesem Artikel nicht um eine qualitative Wertung der vorhandenen Evidenz, noch um die bereits anderweitig beschriebene Rationale zum Einsatz der Hämoadsorption im intensivmedizinischen Setting gehen [2], sondern nur um einen deskriptiven Vergleich zwischen den am Markt erhältlichen Technologien.

Zwischen den verfügbaren Verfahren bestehen teils erhebliche Unterschiede hinsichtlich verwendeter Materialien, Adsorptionscharakteristika, Anwendung und verfügbarer Daten zu Sicherheit und klinischen Erfahrungen. Unserer Ansicht nach sind diese Verfahren – entgegen der teilweise von den Herstellerfirmen (und deren Vertriebspartnern) kommunizierten Behauptungen – in Bezug auf die o. g. Kriterien keinesfalls als gleichwertig und austauschbar zu betrachten.

Wir möchten im Folgenden exemplarisch auf unterschiedliche Aspekte zwischen den am Markt angebotenen Hämadsorptionsprodukten CytoSorb® (CytoSorbents^TM^ Inc., Monmouth Junction, NJ, USA) sowie 2 als Alternativen vertriebenen Verfahren eingehen. Es handelt sich dabei um Hämoadsorber der Jafron® HA-Serie (Jafron Biomedical Co., Guangdong, China) und der Biosky® MG-Serie (Biosun® Medical Technology Co., Foshan City, Guangdong Province, China).

## Aktuelle wissenschaftliche Datenlage

Die Recherche bezüglich der wissenschaftlichen Aktivität in Form von PubMed-gelisteten Arbeiten als für den klinischen Einsatz wichtigstes Kriterium zeigt eine beträchtliche Divergenz zwischen den einzelnen Produkten. Im Fall des HA-330 aus der Jafron HA-Series im Bereich kritisch kranker Patienten fallen lediglich 2 Single-Center-Studien aus dem Jahre 2010 und 2013 auf, die jeweils aus derselben Arbeitsgruppe stammen [15, 16].

Ein weiterer Adsorber (Biosky MG 350 aus der Biosun MG-Series), der aktuell in den Bereichen Intensivmedizin und Herzchirurgie zu vermarkten begonnen wird, kann in diesen Anwendungsgebieten derzeit überhaupt keine spezifische Referenz vorweisen.

Demgegenüber finden sich zur CytoSorb-Technologie, bei ähnlich lang bestehender CE-Zulassung, diverse Arbeiten aus einer Vielzahl von Zentren, die in Peer-review-Journalen veröffentlicht wurden [4, 5, 8–11, 13, 14, 18, 19, 21–24]. Darüber hinaus findet man im bekannten Register für klinische Studien (Clinicaltrials.gov) mit Stand Januar 2020 insgesamt 33 Projekte zur CytoSorb-Technologie und 3 zur Jafron-Technologie, wobei in den gelisteten Studien mit dem HA-330 als Hauptindikation die Behandlung von chronischem Nierenversagen und nicht der Einsatz in der Intensivmedizin ausgewiesen ist. Zu Biosky sind dort bisher keinerlei Angaben zu finden.

Somit ist für die CytoSorb-Technologie zwar deutlich mehr Literatur vorhanden, jedoch ist auch hier die Datenlage vor allem im Hinblick auf harte klinische Endpunkte, wie Intensivaufenthalt, Dauer von Organersatzverfahren und Verbesserung des Überlebens, noch nicht ausreichend und kann daher noch nicht als abgeschlossen angesehen werden.

## Technische Merkmale der Adsorbertechnologien

Auch hinsichtlich des verwendeten Materials und der technischen Spezifikationen unterscheiden sich die Adsorptionstechniken grundlegend (Tab. 1).

**Table 1:** 

	CytoSorb 300 [7] (CytoSorbents^TM^ Inc., Monmouth Junction, NJ, USA)	Jafron HA-330 [17] (Jafron Biomedical Co., Guangdong, China)	Biosun MG350 [1] (Biosun® Medical Technology Co., Foshan City, Guangdong Province, China)
*Adsorptionsmaterial*	Vernetztes Divinylbenzol mit Polyvinylpyrrolidonbeschichtung	Neutrales makroporöses Harz	Neutrales makroporöses synthetisches Harz
*Anwendungszeit pro Adsorber*	24/h bis zu 7 konsekutive Tage	„Die Dauer einer Hämoperfusion ist gewöhnlich 120–150 min“	„Die empfohlene Behandlungszeit mit Hämoperfusion ist 120–180 min. […] Es wird nicht empfohlen, mehr als 3‑mal innerhalb von 24 h zu behandeln“
*Maximaler Blutfluss*	700 ml/min	250 ml/min	400 ml/min

Der CytoSorb-Adsorber enthält Polymer-Beads (vernetztes Divinylbenzol mit Polyvinylpyrrolidonbeschichtung), die Substanzen (vornehmlich hydrophobe) im Bereich bis circa 55 kDa adsorbieren. Dieser Adsorber ist bisher zugelassen für Indikationen, bei denen Plasmaspiegel von Zytokinen, Bilirubin oder Myoglobin erhöht sind. Die Jafron-HA-330-Trägersubstanz basiert auf einem neutralen makroporösen Harz mit Kollodiumbeschichtung. Das Biosun-MG350-Adsorptionsmaterial basiert auf einem neutralen makroporösen synthetischen Harz.

In der Arbeit von Chen und Mitarbeitern zeigte sich, dass das Design der Adsorptionsmaterialien von Hersteller zu Hersteller bezüglich innerer Oberfläche und Größenverteilung der Porenstruktur sehr unterschiedlich ist und Ergebnisse aus vergleichenden Produkttests oder Studien daher nicht von einer auf eine andere Technologie übertragen werden können [6].

Bei den genannten Technologien steht die Elimination von inflammatorischen Parametern, besonders von Zytokinen, im Fokus. Ganz allgemein sind für die als inflammatorische Leitparameter geltenden „Zytokine“ zu CytoSorb, im Gegensatz zu den anderen Adsorbertechnologien, deutlich mehr Daten zu reduzierenden Effekten und klinischen Effekten publiziert. In weiteren, präklinischen Studien konnte darüber hinaus nachgewiesen werden, dass CytoSorb in der Lage ist, ein breites Spektrum von Zytokinen, „damage-associated molecular patterns“ (DAMP), „pathogen-associated molecular patterns“ (PAMP) und Mykotoxinen zu entfernen [12]. Publikationen zum durch die beiden anderen Adsorber entfernten Spektrum fehlen bisher auch im experimentellen Setting. Alle Adsorber nutzen unterschiedliche physikalisch-chemische Effekte, um Substanzen zu adsorbieren. Bei einem Vergleich der Technologien muss daher zwingend auch deren Wirkprinzip betrachtet werden.

## Applikationsspezifische Besonderheiten

Bei der Analyse des Prozesses zur Anwendungsvorbereitung zeigt sich, dass dieser im Fall des HA-330 verhältnismäßig komplex ist und mehrerer Schritte und verschiedener Spüllösungen bedarf, bevor der Adsorber einsetzbar ist. Bei Nichteinhaltung der Prozedur wird explizit auf Hämolyse- und Blutgerinnungsrisiken hingewiesen. Im Fall des Biosky muss ebenfalls mit Kochsalzlösung, jedoch mittels einer Pumpe mit vorgegebener Flussrate gespült werden. Der Spülvorgang bei CytoSorb ist demgegenüber mittels isotoner Kochsalzlösung innerhalb weniger Minuten und ohne weiteres technisches Zubehör durchführbar.

Bei Patienten mit heparininduzierter Thrombozytopenie ist der Einsatz von CytoSorb und Biosky grundsätzlich möglich, während dies bei HA-330 eine Kontraindikation darstellt.

Ein deutlicher Unterschied zwischen den Verfahren tritt auch bei den laut Gebrauchsanweisung abgedeckten Kombinationsmöglichkeiten mit gebräuchlichen extrakorporalen Verfahren zu Tage. Während der HA-330-Adsorber „on-label“ gemäß Angaben in der Bedienungsanleitung lediglich für Hämoperfusion oder Hämodialyse eingesetzt werden kann, erstreckt sich das Spektrum beim CytoSorb-Adsorber neben den genannten zusätzlich auf die Integration in eine Hämofiltration, die Herz-Lungen-Maschine sowie die extrakorporale Membranoxygenierung (ECMO). Die Biosky-Bedienungsanleitung beschreibt den Aufbau mit Hämoperfusion, Dialyse und kontinuierlicher Nierenersatztherapie (CRRT).

Typische Blutflüsse in einem passiven, druckgradientbetriebenen Adsorberbypass an der Herz-Lungen-Maschine und ECMO sind erfahrungsgemäß ca. 12–15 % der Hauptstromblutflussrate. Lediglich CytoSorb ist gemäß Bedienungsanleitung für den Betrieb bis 700 ml/min zugelassen, was sich mit den typischen Bypassblutfüssen deckt [3, 20]. Die anderen betrachteten Produkte sind nur für einen Blutfluss von 200–400 ml/min zugelassen und erfordern entweder den Betrieb über eine Pumpe oder eine Doppler-Flussmessung, um den Fluss auf den zugelassenen Bereich zu limitieren.

## Ganzheitliche Kostenbetrachtung

Als wesentliches Argument zum Einsatz werden bei immer angespannteren Krankenhausbudgets häufig auch die deutlichen Preisunterschiede zwischen den verschiedenen Adsorbern genannt.

In diesem Zusammenhang muss aus unserer Sicht jedoch auch die zugelassene und durch klinische Daten belegte Anwendungszeit unter Berücksichtigung der maximalen Adsorptionskapazität, d. h. sichere und sinnvolle Behandlungsdauer, pro Adsorber in Betracht gezogen werden.

Die maximale Behandlungszeit pro CytoSorb-Adsorber ist mit 24 h ausgewiesen [7]. Die Adsorber von Biosky können maximal 3‑mal täglich mit 120–180 min pro Adsorber eingesetzt werden [1]. Eine Hämoperfusionsbehandlung mit dem HA-330 sollte laut Gebrauchsanweisung in der Regel zwischen 120–150 min dauern [17]. Jafron- und Biosky-Adsorber sind damit bei bestimmungsgemäßer Anwendung im Rahmen der CE-Zulassung zeitlich nur beschränkt und intermittierend einsetzbar, während mit dem CytoSorb-Adsorber prinzipiell eine kontinuierliche Therapie über 24 h an 7 konsekutiven Tagen durchführbar wäre. Aus diesem Grund können bisher auch keine Rückschlüsse auf die wahrscheinlich bestehenden Unterschiede in der maximalen Adsorbtionskapazität getroffen werden.

Sollte eine kontinuierliche Behandlung klinisch sinnvoll sein, wäre dies, zur Vermeidung einer Abweichung von den Spezifikationen der Bedienungsanleitung und somit einer Off-label-Anwendung, mit Jafron- und Biosky-Adsorbern nur mit häufigerem Adsorberwechsel (d. h. höheren Tagestherapiekosten) und erhöhtem Personalaufwand möglich. Dies würde letztendlich einen Kostenvorteil, mit dem die Adsorber von Jafron und Biosky im Markt beworben werden, zunichtemachen oder sogar ins Gegenteil verkehren. Hinzu kommt ein nicht abzuschätzendes Zusatzrisiko für den Patienten durch häufigeres Abhängen des CRRT-Systems und vermehrten Adsorberwechsel (Hygiene, Blutverlust) sowie die potenziellen klinischen Nachteile einer intermittierenden Therapie aus vorher bereits erwähnter Adsorbtionskapazität.

## Resümee

Zusammenfassend bleibt festzuhalten, dass bei den derzeit angebotenen Blutreinigungstechnologien nicht nur deren Wirksamkeit unter Berücksichtigung eines Effekt-Preis-Leistungs-Verhältnisses betrachtet werden sollte, sondern insbesondere auch die Sicherheit, Einfachheit in der Anwendung und Kombinierbarkeit mit anderen indizierten intensivmedizinischen Notwendigkeiten der einzelnen Verfahren von entscheidender Bedeutung ist.

Unter den analysierten Technologien stellt unserer Ansicht nach derzeit CytoSorb das am besten untersuchte und auch klinisch am weitesten etablierte Verfahren dar. Darüber hinaus ist darauf hinzuweisen, dass klinische Ergebnisse, insbesondere jedoch auch sicherheitsrelevante Aspekte, aufgrund der technisch unterschiedlichen Verfahren nicht zwischen den Produkten, wiewohl aus der gleichen Technologiefamilie, übertragbar sind.

## Fazit für die Praxis

- Hämoadsorptionsverfahren zur Blutreinigung bei kritisch kranken Patienten haben in den letzten Jahren klinisch einen zunehmenden Stellenwert erworben.
- Für diese Verfahren sind derzeit mehrere unterschiedliche Produkte im Markt verfügbar.
- Klinische Ergebnisse sowie sicherheitsrelevante Aspekte sind aufgrund der technisch unterschiedlichen Verfahren nicht zwischen den verschiedenen Hämoadsorptionsprodukten übertragbar.
- Es bestehen beträchtliche Unterschiede zwischen den Systemen hinsichtlich der technischen Spezifikationen, der Handhabung sowie der Kompatibilität mit bestehenden extrakorporalen Verfahren.
